# Analysis of tumor template from multiple compartments in a blood sample provides complementary access to peripheral tumor biomarkers

**DOI:** 10.18632/oncotarget.8494

**Published:** 2016-03-30

**Authors:** William M. Strauss, Chris Carter, Jill Simmons, Erich Klem, Nathan Goodman, Behrad Vahidi, Juan Romero, Michael Masterman-Smith, Ruth O'Regan, Keerthi Gogineni, Lee Schwartzberg, Laura K. Austin, Paul W. Dempsey, Massimo Cristofanilli

**Affiliations:** ^1^ Cynvenio Biosystems, Westlake Village, CA, 91361, USA; ^2^ Independent, Seattle, WA, 98109, USA; ^3^ Department of Hematology and Medical Oncology, Winship Cancer Center, Emory University, Atlanta, GA, 30322, USA; ^4^ Division of Hematology/Oncology, University of Wisconsin, Madison, WI, 53792, USA; ^5^ The West Clinic, Germantown, TN, 38138, USA; ^6^ Department of Medical Oncology, Kimmel Cancer Center, Thomas Jefferson University, Philadelphia, PA, 19107, USA; ^7^ Lurie Cancer Center, Northwestern University, Chicago, IL, 60611, USA; ^8^ Current Address: Xencor, Inc, Monrovia, CA 91016, USA

**Keywords:** liquid biopsy, CTC, cfDNA, metastatic breast cancer, next generation sequence

## Abstract

Targeted cancer therapeutics are promised to have a major impact on cancer treatment and survival. Successful application of these novel treatments requires a molecular definition of a patient's disease typically achieved through the use of tissue biopsies. Alternatively, allowing longitudinal monitoring, biomarkers derived from blood, isolated either from circulating tumor cell derived DNA (ctcDNA) or circulating cell-free tumor DNA (ccfDNA) may be evaluated. In order to use blood derived templates for mutational profiling in clinical decisions, it is essential to understand the different template qualities and how they compare to biopsy derived template DNA as both blood-based templates are rare and distinct from the gold-standard. Using a next generation re-sequencing strategy, concordance of the mutational spectrum was evaluated in 32 patient-matched ctcDNA and ccfDNA templates with comparison to tissue biopsy derived DNA template. Different CTC antibody capture systems for DNA isolation from patient blood samples were also compared. Significant overlap was observed between ctcDNA, ccfDNA and tissue derived templates. Interestingly, if the results of ctcDNA and ccfDNA template sequencing were combined, productive samples showed similar detection frequency (56% vs 58%), were temporally flexible, and were complementary both to each other and the gold standard. These observations justify the use of a multiple template approach to the liquid biopsy, where germline, ctcDNA, and ccfDNA templates are employed for clinical diagnostic purposes and open a path to comprehensive blood derived biomarker access.

## INTRODUCTION

Cancer remains one of the leading causes of morbidity worldwide. Treatment decisions and response monitoring is historically dependent on serial imaging technologies and disease-specific pathologic characterization of tissue biopsies typically obtained at time of primary surgery. This generic and standard approach does not support effective cures in the advanced setting primarily because of the inability of cytotoxic therapy to deal with tumor heterogeneity. In the last few years, the emergence of next generation sequencing tools has cast a very different light on the nature of tumor clonality with the ability to build an emerging model describing a much more heterogeneous disease than previously understood [1, 2]. This raises problems in determining the best diagnostic approach when dealing with a disease characterized by a dynamic plasticity that could be not captured in its complexity by the simple molecular snapshot offered by a one-time tissue biopsy. The traditional biopsy is increasingly understood as too restrictive to monitor relevant changes in progression and resistance because it represents a “geographically” and “temporally” restricted sample with implications in patients' management [3]. This has driven the development of various “liquid biopsy” technologies that seek to address the need for monitoring tools that use the specific readout of DNA based biomarkers to monitor changes in tumor profile [4].

The earliest version of a liquid biopsy was built on careful enumeration of a small number of cells that could be found in the blood of patients with epithelial cancers [5, 6]. In the most mature form, this resulted in the FDA approved CellSearch™ test. Using EpCAM to recover, and intracellular expression of Cytokeratin to detect a population of cells, it was shown this population of circulating epithelial cells predict worse outcome, faster disease progression, and increased likelihood of metastatic events [7]. This population came to be known as circulating tumor cells (CTC). However there were several problems demonstrated with the approach. Molecular evaluation has shown some but not all of the cells bear molecular hallmarks of cancer [8-10]. Also, this restricted phenotypic definition of a tumor cell was described prognostically and so excluded many classes of informative cells [11-14]. As a result, CTC enumeration using these legacy definitions frequently returns limited or absent numbers of cells. Maybe most importantly, CTC counting has been ineffective at demonstrating clinical utility in the advanced setting for individual patients [15]. Despite those limitations, because of their detection in a peripheral blood sample, circulating tumor cells provide an attractive source of genetic material for longitudinal monitoring in view of the minimal invasiveness of a blood draw and their potential to reflect the molecular profile of the metastatic cell population [8, 16-17].

Circulating cell-free DNA was identified in 1948 in the plasma and derives from both normal and diseased tissue [18]. Clinical studies have shown ccfDNA can act as a suitable template for cancer monitoring and management [19-22]. Unlike circulating tumor cells, studies suggest ccfDNA representation aligns with disease burden and disease type [23]. As such, the reasons ctcDNA and ccfDNA are found in blood are different with CTC representing the mobile cellular aspect of a tumor while ccfDNA is produced chiefly as a product of apoptosis [24, 25].

In summary, two primary sources of DNA template are available from cancer patient blood samples. These templates have been interrogated for specific known mutations using ultra sensitive PCR based techniques [26]. However the mutational spectrum from these two sample types have never been compared because of the signal to noise challenge for enriching rare tumor cells and a clear comparison of template quality has never been performed. Furthermore, there has not been a concordance study between ctcDNA, ccfDNA, and formalin fixed, paraffin embedded tissue (FFPE) from the same patient and blood draw using sequencing. One reason for this has been technical. Previous studies to compare the two templates have reached different conclusions based on technical limitations for enrichment techniques or based on evaluation with molecular tools that are not directly comparable [21]. Recent advances in separation technologies have provided a solution for this technical problem [27]. The LiquidBiopsy^®^ platform (Cynvenio Biosystems), allows for isolation of useful amounts of ccfDNA and ctcDNA from the same patient blood draw. Typically the amount of ccfDNA and ctcDNA recovered per blood draw is sufficient to produce patient matched NGS libraries. The use of NGS means tumor cells and tumor derived DNA fragments can be defined as mutation bearing events and can therefore be directly compared. In this report we describe a generalizable strategy using NGS on 32 matched FFPE, ccfDNA and ctcDNA samples from clinical samples.

## RESULTS

### Characterization of clinical samples

In this study, patients were recruited based on a confirmed diagnosis of metastatic breast cancer. The samples were collected at baseline, either before start of a new therapy or at completion of staging diagnosis. A tissue biopsy specimen of the metastatic recurrence was mandatory. The patients were predominantly female (97%) and all had stage IV disease. The remaining characteristics are shown in Table 1. Formalin-fixed, paraffin-embedded (FFPE) tumor specimens were obtained for all patients for whom sufficient tissue remained in the pathology block of the metastatic lesion. Of 32 patients, 7 had either insufficient DNA recovered for sequencing, too little or no tumor component to the biopsy or no remaining biopsy tissue. 25 of 32 samples were successfully evaluated.

**Table 1:** Characteristics of normal and metastatic cancer samples analyzed for peripheral templates

Characteristic		Number	Range (%)
**Normal Controls**			
**Number**			
	total Normals	31	
**Age (years)**			
	Median	48	30-74
**Sex**			
	Male	13	(42%)
	Female	15	(48%)
	Unknown	3	(10%)
**Metastatic Cancer Samples**			
**Number**			
	Total	32	
**Age (years)**			
	Median	55	36-82
**Sex**			
	Male	1	(3%)
	Female	31	(97%)
**BC subtype**			
	Luminal A	15	(47%)
	Luminal B	6	(19%)
	Her2	4	(13%)
	TNBC	6	(19%)
	Unknown	1	(3%)
**Pathologic stage**			
	Stage IV	31	(97%)
	Unknown	1	(3%)
**No. of lines of Therapy**			
	Median	4	0-13
**Sampling Interval**			
	Synchronous (≤6 months)	17	0-18 weeks (53%)
	Asynchronous (≥6 months)	14	31-240 weeks (44%)
	Not available	3	(3%)

### Target recovery and extraction of ctcDNA and ccfDNA template

**Figure 1:**
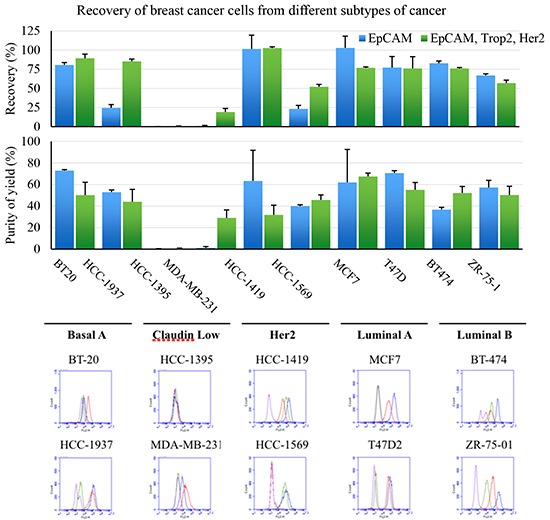
Tumor cells representing the five major subtypes of breast cancer were recovered from 7.5mL blood samples using the LiquidBiopsy system The efficiency of recovery using EpCAM compared to capture with EpCAM, Her2, and Trop2 (top graph) and purity (bottom graph) are shown. Each sample was evaluated in quadruplicate and the bars represent means (+ 1 SD) The same cell lines were evaluated by FACs for control IgG1 (purple), EpCAM (blue), Trop2 (red) or Her2 expression (green) as detected using Streptavidin-FITC.

EpCAM is the legacy marker for recovery of CTC in breast cancer. To expand the definition of cells that can be functionally recovered, a cocktail of antibodies that recognize surface receptors in addition to EpCAM were evaluated. Tumor cells representing the five major subtypes of breast cancer were added to 7.5mL normal healthy blood samples and recovered using the LiquidBiopsy system (Figure 1). The five subtypes; Basal, Claudin low, Her2, Luminal A and Luminal B, were each represented by two different well characterized tumor cell lines [28]. The efficiency of recovery using EpCAM was compared to capture with EpCAM, Her2, and Trop2 (an epithelial/mesenchymal transition profile (EMT)). Recovery (top graph) and purity (bottom graph) of samples engineered with 90c/mL of each cell line were determined. The same cell lines were evaluated by FACs for expression of the three markers. EpCAM only based recovery gave between <1% and 103% recovery of engineered samples. Consistent with patterns of expression, Claudin low and Basal type breast cancer cell lines demonstrated the least recovery with EpCAM alone. Addition of Her2 and Trop2 specific antibodies incremented recovery of target cells when those receptors were expressed. So HCC-1569, MDA-MD-231 and HCC-1937 cell recovery was significantly improved by the pool of antibodies. There is a consistent inhibition of capture of MCF7 cells when the mixture of antibodies is used possibly due to stearic hinderance. Despite this, when a receptor is available for capture, the 90c/mL samples were recovered with an average efficiency of 77% using the EMT cocktail. Critically, with the exception of HCC-1395 which demonstrated no significant capture with either reagent set, 90 tumor cells/mL could be enriched to an average purity of between 29% and 67% from whole blood (see bottom graph). Thus, the LiquidBiopsy platform could reproducibly enrich target cells on the order of 10^5^-10^6^ fold enrichment.

Applying this approach to clinical samples, tumor cell populations were enriched using EpCAM targeted capture and compared to EMT targeted capture. This enrichment protocol was applied to 32 serial patient samples from metastatic breast cancer who were otherwise not selected for elevated CTC numbers. EpCAM based enrichment served as a control and was compared to cell populations selected with EMT cocktail. 32 blood samples were processed for EpCAM only with an average recovered purity of 7.7% Cytokeratin positive (CK+) cells. By comparison, EMT selection enriched populations with on average 8.8% cytokeratin positive cell populations with a larger range of cells than EpCAM selected (see Table 2). Importantly, the median number of CK+ cells recovered from EMT capture was almost three times elevated over EpCAM alone. (median of 23.5 cells/7.5mL vs 8.0 cells/7.5mL for EMT and EpCAM respectively). Also, the median number of CD45+ non-target cells captured was 116 and 172 cells respectively. This background is relevant to a sequencing test that supports detection of mutations present at >1%. ccfDNA sample was purified from the same tube of blood. The average concentration of ccfDNA recovered from fixed plasma samples was 7.3 ng/mL.

**Table 2:** Target cell recovery performance

	CK+ cells/7.5mL		CK+ purity (%CK+)		CD45+/ DAPI+/7.5mL
Capture Cocktail	Median	sd	Average	Range	Median
**EpCAM**	8.0	20.3	7.7%	1 - 112	116
**EpCAM/Her2/Trop2**	23.5	88.4	8.8%	2 - 487	172

### Evaluation of template quality

Interpreting NGS data rests on a clear understanding of the quality of template that is being interrogated. A sequencing pipeline was designed supporting case control detection of single point variants to 1% (Supplementary Figure S1). A primary distinguishing feature between ccfDNA and ctcDNA is the fragmented nature of the ccfDNA that has been described and observed previously [29, 30]. For the purposes of this experiment, the variance of amplification efficiency manifest in the ccfDNA demonstrated that approximately half of the amplicons in ccfDNA amplified the target sequence less efficiently than control (Supplementary Figure S2). To ensure sufficient coverage of the fragmented template, we therefore decided to devote one 318 chip to the analysis of each ccfDNA sample.

A second characteristic distinguishing ccfDNA and cell based DNA sequence came from the evaluation of the SNV substitution frequency (SNV-SF). The SNV-SF results in a quality metric that can be used to evaluate accuracy and precision as well as process-associated or biologically-associated noise and was used as a general metric for mutation frequency. To evaluate SNV-SF, template from the different compartments recovered from a single sample were evaluated using the AmpliSeq hotspot panel. After alignment, the template specific libraries were compared to germline sequence and only alterations with >2000 reads were evaluated. However, the reportable range for SNV-SF determination was not restricted to the Cosmic identified SNV in the Hotspot library. Therefore, any alteration detected that could not be eliminated by comparison to germline was included. This general calculation gives a comparison of different sample types from the same patient.

**Figure 2:**
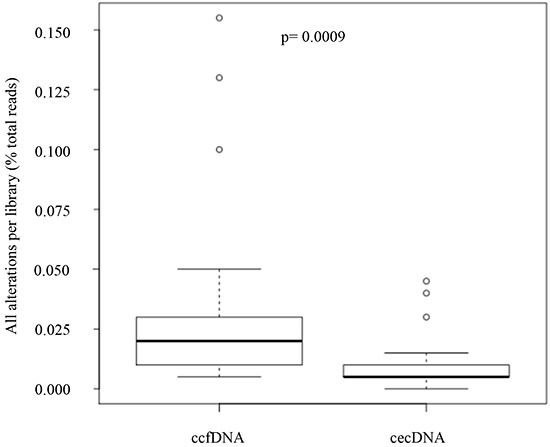
Evaluation of template noise in cell free DNA and cells enriched from blood as measured by SNV-SF Circulating epithelial cell DNA (cecDNA) or matched cell free DNA was recovered from 29 normal healthy donors Paired template were sequenced using the AmpliSeq library and compared to germline sequence from the same sample. Alterations with an extended reportable range to include all evaluated bases in the library were enumerated. A two-tailed Wilcoxon rank sum test measured a mean difference of 3.0 and a p value of 0.0009.

The SNV-SF was calculated on ccfDNA and circulating cell DNA recovered from 29 normal healthy volunteers. This analysis revealed a three-fold difference in the number of alterations observed in a known negative sample set (Figure 2). Thus, there is a noise variable manifest in ccfDNA as altered sequence that is based on the compartment of the DNA template rather than the sequencing reaction or the sequencing platform. A two-tailed Wilcoxon rank sum test measured a p value of 0.0009. If we assume that all SNVs detected in healthy donors are false positives then there is a 3-fold higher noise in the ccfDNA samples compared to the matched circulating cell DNA sample. This difference is detected on template fixed in the same sample tube, for the same time and measured with the same test. It therefore suggests the noise variable in ccfDNA is a biological noise rather than a system or assay noise. As a result, the primary comparison of ccfDNA and ctcDNA was restricted to the panel of COSMIC validated SNVs mapping to the AmpliSeq Hotspot panel in the CLIA validated test. These restrictions allowed *de novo* identification of template associated mutations across >2500 different mutations and therefore represents a toolset and workflow that supports mutation discovery on multiple templates from a single blood draw.

### Clinical sequence output: Primary comparison of different templates

An important measure for clinical relevance of a NGS test is the detection of disease associated alterations in templates derived from tumor sample. This experiment was conducted on a cohort of metastatic breast cancer samples. After assembly, all variants were filtered to yield COSMIC validated mutations. For cell enrichment, we initially included EpCAM based recovery to compare capture to a cocktail of EpCAM/Her2/Trop2. As shown in Supplementary Table S1, the frequency with which EpCAM capture alone supported identification of mutation bearing cells was 9%. The EMT cocktail outperformed the EpCAM only capture by 3-fold (Table 3). Therefore, these data focus on characterizing the EMT performance.

**Table 3:** COSMIC identified SNV from matched tumor samples derived from blood or biopsy

ID	E/P/H	ctcDNA	ccfDNA	FFPE 1	FFPE 2
C293-001	+/+/−	X	X	X	
C293-002	−/−/+	TP53; p.E285K	X	QNS tissue	
C293-003	−/−/+	X	X	X	
C293-004	+/−/−	X	PIK3CA;p.E542K	PIK3CA;p.E542K	PIK3CA;p.E542K
C293-005	+/−/+	X	PIK3CA; p.V344G ERBB2; p.V777L	PIK3CA; p.V344G ERBB2; p.V777L	
C293-006	−/−/−	X	TP53;p.H193R	TP53;p.H193R	
C293-007	−/−/−	X	X	KRAS; V14I	
C293-008	NA	X	TP53; p.S215R	QNS tissue	
C293-009	+/+/Eq	X	X	X	
C293-010	+/−/+	TP53; p.Y163D	TP53; p.Y163D	QNS Tissue	
C293-011	−/−/−	X	TP53; p.R175H	TP53; p.R175H	
C293-012	+/−/−	X	X	X	
C293-013	+/+/−	X	QNS DNA	PIK3CA; p.H1047R	X
C293-014	+/+/Eq	X	TP53; p.M246I	X	
C293-015	−/−/−	X	X	X	
C293-016	−/−/+	PIK3CA; p.E545K	X	QNS Tissue	
C293-018	+/−/−	PIK3CA; p.H1047R	PIK3CA; p.H1047R	QNS DNA	PIK3CA; p.H1047R
C293-019	+/+/−	X	X	IDH2; p.R140Q	
C293-020	−/−/−	X	X	X	
C293-021	+/+/−	X	GNAQ; Q209K	X	
C293-022	+/+/−	X	X	PIK3CA; p.E545K	
C293-023	−/+/Eq	TP53; p.R175H	TP53; p.R175H	TP53; p.R175H	
C293-024	+/−/−	X	PIK3CA; p.H1047R	X	PIK3CA; p.H1047R
C293-025	+/−/−	X	X	X	
C293-026	+/−/Eq	PIK3CA; p.H1047R	PIK3CA; p.H1047R	TP53; p.C182Y	APC; p.Q1447* TP53; p.G108S PIK3CA; p.H1047R
C293-027	+/+/−	TP53; p.H178P	X	X	
C293-028	+/−/−	X	X	QNS DNA	
C293-029	−/−/+	X	TP53; p.E286K	QNS DNA	
C293-030	+/+/−	X	X	ERBB2; V777L	
C293-031	+/+/−	X	X	X	
C293-032	−/−/−	X	TP53; p.Y107*	X	
C293-033	+/+/−	TP53; p.C176F	TP53; p.C176F TP53; p.C176S	TP53; p.C176F	

Most recent hormonal (ER/PR) and Her2 status is included where available (E/P/H) and indicated as present (+), absent (-), or equivocal (Eq). Alterations are indicated by the target gene and the predicted impact. Samples with no detectable alteration at the limit of detection are indicated by X. Samples that were quantity not sufficient (QNS) are indicated.

The ctcDNA and ccfDNA samples were analyzed using a case-control model with a limit of detection of 1%. For FFPE, no case-control was used and the limit of detection was 10%. The incidence for detection of variants in evaluable FFPE samples using the AmpliSeq panel was 54% (14 of 26 with 6 samples QNS). The genes most frequently mutated were *TP53* and *PIK3CA* with mutation frequencies of 20 and 28% respectively. This is consistent with these being the most frequently altered genes in breast cancer. The frequency of mutations observed in ccfDNA and EMT ctcDNA samples was 48% and 25% respectively. Similar to the FFPE analysis, the most frequently altered genes were again *TP53* and *PIK3CA*. Specifically, mutations in *TP53* and *PIK3CA* were detected with 16% and 9% frequency respectively in the ctcDNA from 32 evaluated samples. Mutations in the same genes were observed with 29% and 16% frequency in ccfDNA. In combination, ccfDNA and EMT ctcDNA produced SNV information 56% of the time - a frequency directly comparable to the FFPE sample frequency of 58%. Therefore overall, the peripheral multitemplate analysis produces signal with 98% of the frequency of evaluable FFPE samples.

The impact of sampling on heterogeneity can be observed both within a sample type as between compartments. For instance, sample CYN-026 described a *TP53* (C182Y) mutation in a bone marrow derived biopsy sample. A synchronous bone marrow sample displayed a distinct *TP53* alteration (G108S) as well as alterations in *APC* (G1447*) and *PIK3CA* (H1047R). The *PIK3CA* alteration but neither of the *TP53* or the *APC* alterations were detected in the peripheral samples. Furthermore, the *PIK3CA* alteration was detected in the EMT population but not the epithelial population. In another sample (CYN-003) a mutation in *PIK3CA* (E542K) is detected in two biopsy samples and the ccfDNA compartment but is not detected in either the epithelial or EMT cells. In contrast, CYN-016 identifies a *PIK3KCA* (E545K) driver mutation in EMT cells that is not detected in ccfDNA. The biopsy sample for the latter subject had insufficient tumor tissue on pathology review for sequence analysis. Thus, whether sampling different biopsy sites or different peripheral compartments, the three different templates are complementary.

Evaluating the impact of different tumor sampling mechanisms has been challenging due to limited studies capable of making direct comparisons between samples and indexing the template quality and performance on different templates but with the same assay. Due to the impact of insufficient biopsy material (QNS), mutations were described in 47% of the FFPE samples (26 evaluable samples of 32 subjects). Of that number, 9 samples (28%) produced peripheral signal from either ccfDNA or ctcDNA or both that was concordant with FFPE. Separately, while 72% of the samples have some information in one compartment or the other, 44% (10 of 23 samples) of any alteration could be confirmed in an orthogonal template (Table 3).

**Figure 3:**
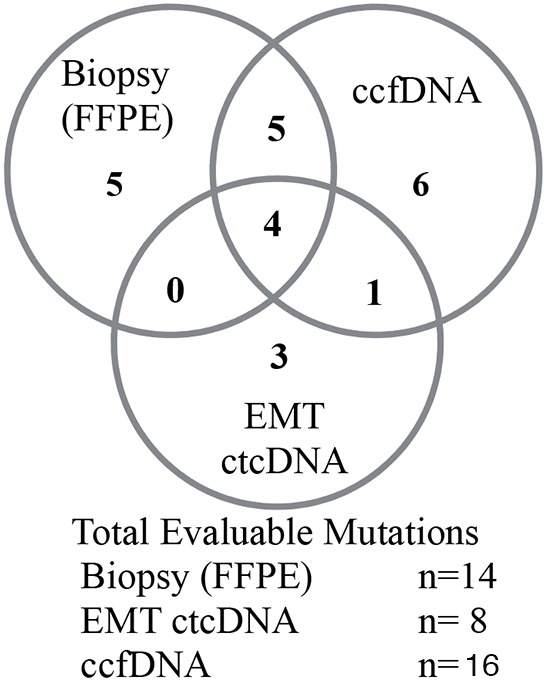
COSMIC identified SNV from matched tumor samples derived from blood or biopsy Alterations are counted in all clinical samples. Overlap between each template is depicted by Venn diagram with comparison between FFPE, ccfDNA and ctcDNA compartments.

The relationship between the different sampling mechanisms is described in Figure 3. The orthogonal nature of the three sampling mechanisms detected is impacted both by the different source of sample as well as the chance that no tumor derived material is detected in either FFPE or the peripheral samples. The overlap of SNV mutation calls between sample types (FFPE, ctcDNA, ccfDNA) was compared. Concordant results were measured as the presence of any specific mutation between different sample types in the same patient. There were clinically relevant and potentially actionable mutations detected in EMT ctcDNA samples or ccfDNA samples that were not shared with other sample types. Thus EMT ccfDNA and ccfDNA produce complementary data that can supplement rare signals in either compartment or can serve as orthogonal confirmatory templates. Furthermore, the ability to tune the informative cell population can dramatically alter the frequency of informative reads.

## DISCUSSION

In this prospective study, we compared NGS data sets from three distinct patient-matched samples types (FFPE, ctcDNA, ccfDNA). A common amplicon based resequencing panel (Ampliseq v2 HotSpot panel) was used for all template types in a NGS pipeline using identical variant analysis. Patients with available quality tissue specimen from diagnostic biopsy were isolated and compared. We demonstrated that ccfDNA and ctcDNA evaluation yields complementary molecular information from the same blood sample. Moreover, the capture of CTC for molecular analysis may be tuned based on a molecular detection system. In fact, the capture protocol for circulating mutation bearing cells was a novel cocktail of anti-EpCAM, Her2, and Trop2 antibodies. This cocktail was designed based on the established role of EpCAM in defining a population of cells in blood that is prognostically related to disease progression as well as metastatic events [31, 32]. The additional markers have established an emerging utility for antibody directed therapies in breast cancer targeting Her2 and Trop2 respectively [33-36]. While other cocktails have been tested, only a subset has been shown to be productive for the detection of mutation bearing cells [11, 37]. Furthermore, in our study the detection and molecular analysis of CTCs was not dependent on clinical or biological outcome to interpret the value of the populations. Specifically, the CellSearch test is not dependent only on EpCAM expression per se. Rather, if anything changes, including cells or reagents, the approved prognostic value is lost. For these applications, the most important result is the demonstration that the informative template from a blood sample may now be defined molecularly as related to the disease process by orthogonal characterization of DNA alterations. DNA based analysis of SNV was utilized to demonstrate qualitative characteristics between the different template compartments. Having validated the capture definition, the informative cell population may now be interrogated for additional biomarkers of value such as expression of protein markers [38], protein modification events, or RNA expression based analysis for biomarkers that are opaque in the DNA compartment such as the AR-V7 splice variant in prostate cancer [39].

The additional goal of these comparisons was to evaluate different template compartments for the ability to provide clinically useful DNA sequence information. The measures of success for these comparisons were deemed to be i) the frequency of disease associated alterations observed in the analysis of multiple templates (tissue, ctcDNA or ccfDNA) and ii) the quality of template for supporting sequence analysis. A key observation is that the FFPE sample and the complementary peripheral samples together provide molecular information in a similar proportion of samples. In these data, all templates are detecting alterations within a two-fold range of each other. Previous studies comparing CTC and ccfDNA have been limited by making comparisons with different technologies [21] or using shared readouts that do not alter the significant signal to noise challenge of detecting rare mutation bearing cells [23]. These approaches made it challenging to perform a direct comparison of ccfDNA and ctcDNA. A recent report demonstrating the presence of a T790M resistance mutation in lung cancer supports the data presented here in that ctcDNA and ccfDNA produce complementary information [26].

Fundamental to this experiment is the ability to clarify the population of cells molecularly. There are numerous approaches to recovering tumor cells from blood samples ranging from size based selection, to mechanical mechanisms, charge based approaches or no selection. Each has applications for discovery of previously uncharacterized cell populations. In a patient setting, population based (as opposed to single cell) analysis of cells or ccfDNA fragments allows more rapid molecular analysis but also uses a sampling mechanism that may be less sensitive to analytic variables. A significant advantage of positive selection approaches is the ability to standardize the enrichment definition around a standardizable biological definition.

The studies reported here are based upon the examination of cell pools, as well as mixtures of ccfDNA isolated from whole blood. To the best of our knowledge, there are no published data that show a population of tumor cells in blood can be productively recovered and sequenced directly yielding a clinically relevant mutational spectrum. Other direct evidence for the significant presence of mutation bearing cells has come from single cell experiments or single cell based readouts such as FISH analysis [10, 40-42]. Despite that, a number of studies demonstrated the presence of cells in blood that contain mutations that can be tracked back to the tumor but almost all are restricted single cell selection [43], single cell analysis [44, 45], or single cell propagation steps [11, 37]. The population based approach we present in this paper supports high throughput based analysis. In addition, a median background of 172 CD45+ events from a 7.5mL blood sample will allow detection of as few as 3-4 heterozygous events from a blood sample using a test that can detect mutations with a 1% frequency; a performance already approaching a single cell detection event. Furthermore, individuals without cancer exhibit recoverable cells with a phenotype related to circulating epithelial cells [46, 47]. However these cells do not bear cancerous SNV mutations. It will be important therefore to develop advanced validation tools, like detection of DNA alterations, before further characterization of cell populations with tools such as expression profiling. Even enumeration has to be indexed to a clinically prognostic impact or molecular demonstration that the cell population is truly tumor derived.

The current data demonstrate that both blood derived templates support informative amplicon based resequencing. However, significant differences between the templates with respect to their performance in NGS variant analysis suggest that the templates need to be handled according to their strengths and weaknesses by standardizing the analysis rules. Key among these observations was the demonstration of a variant noise detected in the ccfDNA that is significantly elevated over cell based analysis from the same sample. This noise was detected at random sites within the amplicon that are not associated with the disease process and need to be eliminated by a restriction call using bioinformatic methods. This has implications for the ability to use this approach to map *de novo* mutations discovered in the ccfDNA template. One hypothesis to explain this noise, postulates that noise is due to the fixative or preservative utilized, analogous to the template damage observed with FFPE template [48]. However the absence of a similar noise profile in other matched DNA templates recovered from the same sample argues against this hypothesis. ccfDNA is a degradative product of catabolism, present in the extracellular compartment and therefore is susceptible to multiple processes of damage in plasma. Indeed, when specifically evaluated, a similar background has been described elsewhere [49]. Alternatively, given the data suggesting accumulation of DNA damage over time in preneoplastic tissues, we can speculate that this template associated noise might be a reflection of that larger phenomenon in biology, in essence detecting a level of “normal” DNA damage even in the absence of diagnosed malignancies with potential implications for future studies [50-52]. Certainly, this difference between ctcDNA and ccfDNA quality reflects the known differences in their biological source and has implications for the prognostic value of each biomarker [53].

Clearly, detection of tumor related events depend on the targets included in the NGS test. The AmpliSeq v2 Hotspot panel is a well curated pan-cancer panel that has been shown to identify disease associated alterations in FFPE and ccfDNA [54-56]. The most frequently mutated alterations in breast cancer are detected with related frequencies in the multiple templates. However, not all disease associated mutations can be included in any targeted panel. More comprehensive whole genome or whole exome sequencing is possible but should be considered in the context of the enzymatic amplification steps necessary to produce sufficient template from limiting numbers of cells and the impact on sequence integrity. Therefore, it will be key to develop detection tools that reflect the most relevant decision points for any given patient population. For instance, in breast cancer, there are clinically relevant biomarker targets in the protein, RNA or DNA compartments [57-63]. Functional access to both ccfDNA and CTC based templates supports development of protein and RNA based readouts in addition to detection of DNA based alterations.

This study demonstrates that it is possible to productively interrogate both populations of mutation bearing cells enriched from a blood sample and ccfDNA. Importantly, this analysis does not require *a priori* knowledge of the mutations present and will serve as a useful discovery tool. Expanding the definition of informative cell populations may now be indexed to a molecular definition. This ability to define populations beyond the legacy epithelial definition raises the possibility of informative biomarkers in non-epithelial settings such as soft tissue cancers, skin cancers such as melanoma, or epithelial cancers that have not previously performed well with strictly epithelial markers but still characterized by metastatic events.

Fundamentally, tumor derived samples from biopsy, circulating tumor cell populations and ccfDNA are acquired by different sampling mechanisms; mechanisms that capture different moments of the disease and potentially not necessarily the same biological sources. The study of these alternate templates by next generation sequencing technologies has emphasized the clonal nature of cancer and the impact of evaluating these different biopsy sites [64]. The emerging model for the peripheral templates suggest ccfDNA samples the genomic DNA fragments released from all tumor sites. In contrast, circulating tumor cells are clearly related to the disease process, predict more aggressive disease and increased metastasis [65]. As such, CTC reflect the mobile and metastatic subset of tumor cells. Clearly the overlap between detectable alterations in each compartment demonstrates the relevant information may not always be linked to one specific compartment or one biology. An ability to evaluate multiple compartments, both in terms of sensitivity and biomarker definition, is an innovative approach that can have significant impact with regards to diagnosis and on our capacity to better understand the various biological processes driving metastases and potentially different therapeutic approaches. The impact of these biological differences will have to be determined in prospective studies.

## MATERIALS AND METHODS

### Ethics statement

This prospective clinical trial was funded with a NIH/NCI contract with the express purpose of expanding the definition of evaluable tumor cells in blood. The long term goal of the SBIR contract was to develop new devices and methods of CTC detection with a focus on clinical tools rather than research based approaches. Patients were enrolled at three different sites (Kimmel Cancer Center of Thomas Jefferson University, Winship Cancer Institute of Emory University, and The West Clinic). Normal human donor blood was purchased from HemaCare Corporation. All subjects provided written informed consent under an Institutional Review Board approved protocols.

### Study subjects

The study was designed to enroll subjects with a confirmed diagnosis of metastatic breast cancer that were about to start a new line of therapy for their disease. A tissue biopsy of the metastatic lesion was mandatory. After appropriate consent enrolled subjects provided a blood sample before starting the new therapy. The blood samples had a minimum volume of 16mL minimum from two K_2_-EDTA tubes with minimal signs of hemolysis. Samples were processed for recovery of CTC populations within 96 hours using a CLIA approved process. Thirty-two metastatic breast cancer patients were recruited. FFPE and blood was recovered from each donor. From the blood samples, germline white blood cell DNA (wbcDNA), ccfDNA, and ctcDNA were isolated. Sequencing libraries were constructed from the matched samples and subjected to NGS using the IonTorrent PGM platform.

### Template enrichment procedures

All sample processing, sequencing and analysis was performed in the Cynvenio Biosystems CAP approved facility (Westlake Village, CA) under CLIA supervision. Whole blood was collected in purple top (K_2_EDTA) tubes and stabilized using LiquidBiopsy (Cynvenio Biosystems) fixative. A white blood cell control was recovered from 0.1cc of the original sample. Plasma was collected after brief centrifugation to separate cellular components. CTC's were enriched as described [25]. In brief, the cellular component in the starting blood volume was blocked with FcR block and labelled with a biotinylated antibody cocktail consisting of anti-EpCAM alone, or in combination with anti-HER2, and anti-TROP2 (Cynvenio Biosystems) followed by iMAG streptavidin beads. The labeled blood was processed in the CTC flow cell on the LiquidBiopsy platform (Cynvenio Biosystems). Captured cells were characterized by evaluating immunofluorescent staining with anti-Cytokeratin, anti-CD45 and DAPI. Captured cells were recovered by centrifugation to produce an enriched cell pellet. The CTC pellet was digested as described [25] and the resulting digest was diluted to 12μL with TE. The AmpliSeq library reagents were added directly to the template and further processed as for the germline and ccfDNA samples.

For enrichment of ccfDNA from the recovered plasma, the QIAamp Circulating Nucleic Acid Kit was used along with the QIAVAC system as recommended by the manufacturer (Qiagen, Valencia, CA). DNA resulting from this purification was quantitated on a Nanodrop (Thermo Fisher Scientific) and directly utilized for sequencing library generation. ccfDNA libraries were produced with a 10 ng input of ccfDNA.

FFPE processed slides were H&E stained, graded by pathologist to indicate the area of specific tumor tissue, macro-dissected, and placed into individual tubes. Three to six 5μm thick sections were processed using the Agencourt® FormaPure® kit (Beckman Coulter). Samples were eluted in 45μL and DNA was concentrated further with Agencourt AMPure XP beads to yield a final DNA volume of 15μL. DNA concentration was measured by Qubit® dsDNA HS (High Sensitivity) Assay Kit (Thermo Fisher Scientific).

### Sequencing data analysis

Primary sequence was demultiplexed and exported from the Torrent Server as FASTQ. The FASTQ files were aligned by reference guided assembly to NCBI GRCh37 p5 using Bowtie 2 [66]. Post-assembly alignments were piled and curated for accuracy using SAM Tools (version 0.1.19) [67] and transferred to Perl using Bio::DB::Sam. ctcDNA and ccfDNA templates were analyzed and a mutation was called if ≥20 mutant reads were observed for a limit of detection (LOD) of 1%. FFPE templates were analyzed to a LOD of 10%. ctcDNA and ccfDNA analysis was based upon a case-control model for variant detection, in which total read coverage must be ≥ 2000 reads per amplicon for ctcDNA and ccfDNA sample and validated calls were required to be absent from the negative control wbcDNA sequence (Supplementary Figure S1). FFPE analysis was not case-controlled, and total read coverage threshold was ≥ 500 reads per amplicon.

## SUPPLEMENTARY METHODS FIGURES AND TABLE


