# Supplementary material for: Analysis of tumor template from multiple compartments in a blood sample provides complementary access to peripheral tumor biomarkers
Source: Oncotarget. 2016 Mar 30;7(18):26724–38. doi: 10.18632/oncotarget.8494 (PMC5042010; doi:10.18632/oncotarget.8494)
Supplement: Supplementary file 1 [file oncotarget-07-26724-s001.pdf]

SUPPLEMENTARY METHODS

Library preparation and ion torrent sequencing

Primary library construction was performed with either ccfDNA, ctcDNA, or wbcDNA and AmpliSeq Library kit v2.0 according to the manufacturers instructions. The reaction contained ≤1ng template. Samples were barcoded and purified on Ampure beads. The quantity of libraries was assessed using the Qubit 2.0 Fluorometer (Thermo Fisher Scientific).

The Ion AmpliSeq™ Cancer Hotspot Panel v2 is a single pool of primers consisting of 207 amplicons spanning 50 gene. It is used to perform multiplex PCR for preparation of amplicon libraries from genomic regions that are frequently mutated in cancer. The library base is ~33 kb and surveys ~3 x 10^3 COSMIC identified mutations. Following library construction, and using manufacturers best practice, libraries were barcoded, purified, and used for ISP emulsion formation. Amplified samples were quantitated using Ion Library Quantitation Kit (Thermo Fisher Scientific). ISP pools consisted of three barcoded libraries ccfDNA/ctcDNA/wbcDNA were then sequenced. ctcDNA and wbcDNA were co-sequenced on the same 318 chip using the IonTorrent PGM (Thermo Fisher Scientific). For each matched ccfDNA sample, due to the low template quality, samples were sequenced using a single dedicated 318 chip. Similarly each FFPE sample was sequenced on a single 318 chip.

Comparison of the template for amplification

ccfDNA and ctcDNA are radically different types of template, despite being derived from whole blood. Both templates offer the possibility of utility for clinical diagnostic purposes. In order to understand the relative value of each template, it was important to understand the quality of the two templates and how it impacts what information can be gathered and how they should be evaluated. Thus one goal of this study was to compare subject-matched ctcDNA and ccfDNA templates by NGS. In order to report a fair comparison, three different qualities of the DNA templates were used. The first quality was "amplicon performance" which is defined to mean how well a particular DNA template supported PCR amplification with primers spanning amplicons from the Ion AmpliSeq Cancer Hotspot Panel v2 which contains multiplexed primers for 207 amplicons. Performance for all amplicons were evaluated for a cohort of known negative normal healthy donor samples which were processed for recovery using the same EpCAM enrichment of circulating epithelial cell derived DNA (cecDNA). Comparative analysis was performed after normalizing either cecDNA or cell free DNA (cfDNA) amplicon-performance results to subject-matched germline wbcDNA control. 29 subjects were compared (Figure 2). Amplicons are presented arranged by size with the smallest amplicons on the left and the largest amplicons on the right. As the amplicon size increases, the efficiency of amplicon generation relative to the germline control diminishes. The variance for ctcDNA samples was fairly consistent across all amplicon sizes. However for cfDNA samples the variance for larger amplicons is very low, consistent with very low amplicon coverage in the larger size range. This is a consistent with the well known fragmented nature of the ccfDNA template [1, 2]. For the purposes of head to head comparison of ccfDNA and cecDNA, these data demonstrate that approximately half the amplicons in cfDNA amplify the target sequence less efficiently than control. Therefore, for a 1% threshold sequence analysis, additional sequencing capacity has to be applied to the analysis of cfDNA.

For a DNA source to be useful in clinical testing, it must support unbiased analysis. To a first approximation, in a NGS test, PCR amplification, as judged by amplicon amplification performance is a test of uniformity. A powerful NGS test should not exhibit significant amplicon to amplicon variation or biasing. In the data presented, biasing is observed for ccfDNA. That biasing is not simply a function of amplicon size is shown clearly in Figure 2. Here the variance between identical amplicons in different samples is substantial. ctcDNA templates show very low variance across all amplicons and sizes. The amplicon performance variance for subject matched ccfDNA samples is quite broad. The smaller amplicons show very large differences in variance while the most under-represented large amplicons show very little variance. That the variance for the larger amplicons is low is not surprising as they perform poorly. What is notable is that "well" performing small amplicons exhibit a large variance. That the smaller amplicons exhibit such template variance suggests that quantitative tests (like CNV) using amplicon performance may have significant technical challenges, especially in reproducibility of measurements due to confounding factors.

REFERENCES

1. Gormally E, Caboux E, Vineis P, Hainaut P. Circulating free DNA in plasma or serum as biomarker of carcinogenesis: practical aspects and biological significance. Mutat Res. 2007; 635:105–117.
2. Wang M, Block TM, Steel L, Brenner DE, Sum Y-H, Preferential isolation of fragmented DNA enhances the detection of circulating mutated k-ras DNA. Clinical Chemistry. 2004; 50;211–213.

A. **Linearity of mutant calls across all chromosomes**

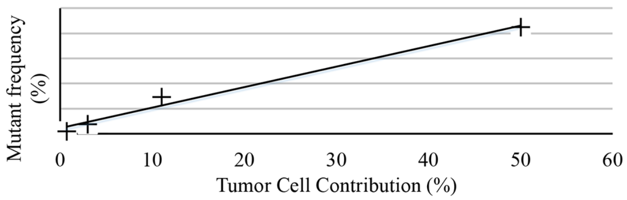

B. Mixed tumor cell lines used for sequencing validation

Mutant Cells	Gene	Mutation	Ploidy	CHR	Scorable
A549	KRAS	G12S	HOM	12	YES
A549	STK11	Q37*	HOM	19	YES
MCF-7	PIK3CA	E707K	HET	3	NO
MCF-7	PIK3CA	E545K	HET	3	YES
HCC1419	TP53	p.A74fs*47	HOM	17	NO
HCC1419	TP53	p.Y220C	HOM	17	YES
H1975	CDKN2A	p.E69*	HOM	9	YES
H1975	EGFR	p.L858R	HET	7	YES
H1975	EGFR	p.T790M	HET	7	YES
H1975	PIK3CA	p.G118D	HET	3	NO
H1975	TP53	p.R273H	HOM	17	YES

C. Performance of CLIA validated PGM based sequencing test

Parameter	Result
Linearity	Linear Range: 0.12% - 100% Allele Frequency<br>R^2 = 0.9925, Lower Limit of Detection: 0.12% (Table 7a, 7b Figure 2)<br>*NOTE: Standard procedure allows a linear range from 1 % - 100 %.*
Sensitivity	0.12% Allele Frequency, SD: 0.823
Reference Range	Human Genome: patch5 of Human Reference assembly GRCh37 derived from National Center for BioInformatics (NCBI)
Intra-Assay Precision	100% Precision
Inter-Assay Precision	100% Precision
Accuracy	100% Accuracy
Reportable Range	All COSMIC validated SNVs mapping to the Life Technologies Ion Torrent Ampliseq 2.0 SNP Panel
Analytical Specificity	100% Specificity

Supplementary Figure S1: Sequencing validation to validate the sequencing readout, we evaluated sensitivity and specificity of the AmpliSeq HotSpot Panel v2 on cohorts of engineered pellets **A.** Pellets were composed of a cell line lacking mutations in the evaluated region (GM12878) engineered with incremental numbers of mixed tumor cell lines (MCL). The admixture of cells included 8 known point mutations **B.** The pellets were engineered with a linear range between 0.75% and 50% MCL representation. The pellets were sequenced in triplicate using GM12878 cells as a germline case control sequence. Using this approach, the sequencing was shown to be 100% sensitive to alterations present at greater than 1% representation. Furthermore the false positive rate was indexed as 0.0015% across all 20000 bp of the library. Reportable range was COSMIC identified alterations in the Ampliseq panel **C.**

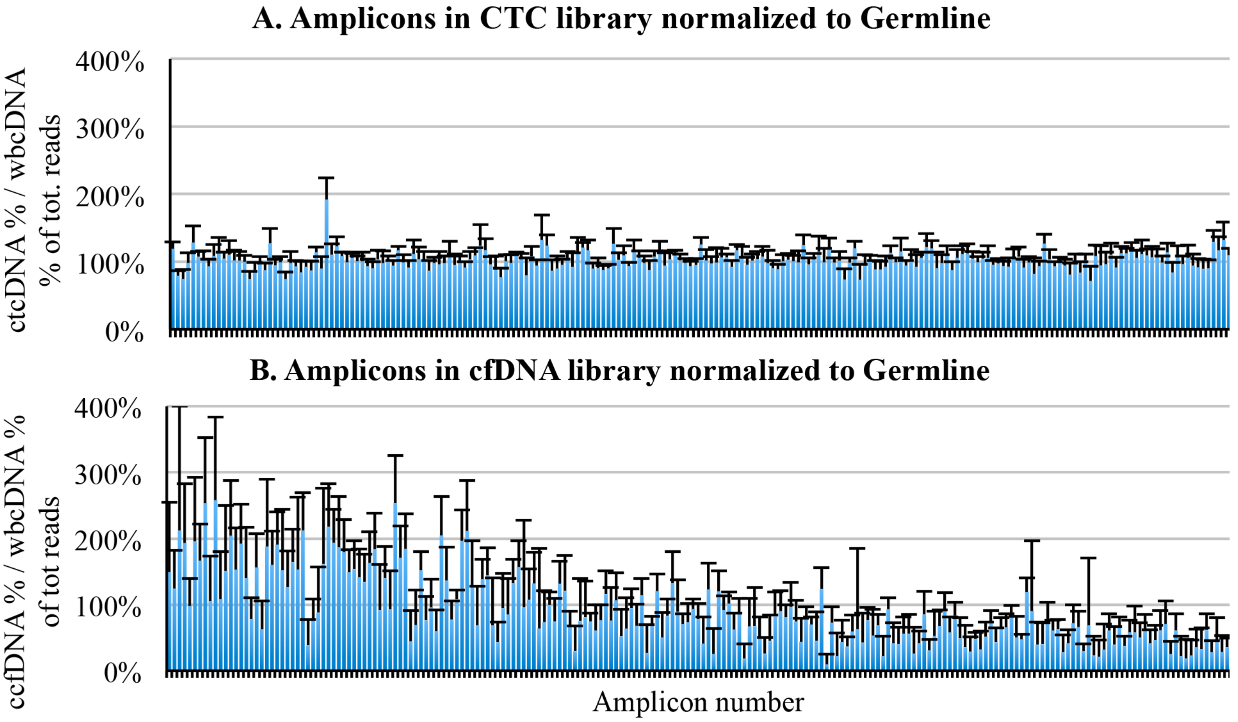

Supplementary Figure S2: Comparison of amplicon performance for cell based and cell free based nucleic acids: Amplicons in CHPv2 libraries built with cecDNA **A.** and cfDNA **B.** from normal healthy controls were compared to germline controls libraries for efficiency of amplification. The graph represents percent efficiency compared to germline control sequence +/− 1 SD across all 207 amplicons arrayed by size (in base pairs). 12 libraries were compared in this analysis but are representative of 29 normal donor samples.

Supplementary Table S1: Epithelial CTC sequencing: COSMIC identified SNV from matched tumor samples derived from EpCAM selected blood or FFPE biopsy

ID	Epcam ctcDNA	FFPE 1	FFPE 2
C293-001	X	X	
C293-002	X	QNS tissue	
C293-003	X	X	
C293-004	X	PIK3CA;p.E542K	PIK3CA;p.E542K
C293-005	X	PIK3CA; p.V344G	
		ERBB2; p.V777L	
C293-006	X	TP53;p.H193R	
C293-007	X	KRAS; V14I	
C293-008	X	QNS tissue	
C293-009	X	X	
C293-010	TP53; p.Y163D	QNS Tissue	
C293-011	X	TP53; p.R175H	
C293-012	X	X	
C293-013	X	PIK3CA; p.H1047R	X
C293-014	X	X	
C293-015	X	X	
C293-016	X	QNS Tissue	
C293-018	PIK3CA; p.H1047R	QNS DNA	PIK3CA; p.H1047R
C293-019	X	IDH2; p.R140Q	
C293-020	X	X	
C293-021	X	X	
C293-022	X	PIK3CA; p.E545K	
C293-023	X	TP53; p.R175H	
C293-024	X	X	PIK3CA; p.H1047R
C293-025	X	X	
C293-026	X	TP53; p.C182Y	APC; p.Q1447*
			TP53; p.G108S
			PIK3CA; p.H1047R
C293-027	X	X	
C293-028	X	QNS DNA	
C293-029	X	QNS DNA	
C293-030	X	ERBB2; V777L	
C293-031	X	X	

(*Continued*)

ID	Epcam ctcDNA	FFPE 1	FFPE 2
C293-032	X	X	
C293-033	TP53; p.C176F	TP53; p.C176F	

Alterations are indicated by the target gene and the predicted impact. Samples with no detectable alteration at the limit of detection are indicated by X. Samples that were quantity not sufficient (QNS) are indicated.
